# Reply to Johansson ‘Treatment with BRICHOS domain helps to clarify issues with Alzheimer mouse models’

**DOI:** 10.1038/s44321-024-00042-0

**Published:** 2024-03-13

**Authors:** Luciano D’Adamio

**Affiliations:** Department of Pharmacology, Physiology & Neuroscience New Jersey Medical School, Brain Health Institute, Jacqueline Krieger Klein Center in Alzheimer’s Disease and Neurodegeneration Research, Rutgers, The State University of New Jersey, 205 South Orange Ave, Newark, NJ 07103 USA

**Keywords:** Neuroscience

## Abstract

This correspondence is a reply to correspondence from Dr. Jan Johansson on the utility of BRICHOS domains in understanding pathology in Alzheimer’s disease mouse models.

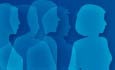

I want to thank Dr. Johansson, who is leading groundbreaking research on the anti-amyloidogenic effects of the BRICHOS domain of BRI2, for this is stimulating letter. To clarify, my commentary (D’Adamio, 2023) was not meant to either rebut or endorse the Amyloid cascade hypothesis and its various iterations. In my opinion, given the current state of knowledge, making an absolute assertion that Aβ (and tau tangles) are entirely unrelated to Alzheimer’s pathogenesis would be just as flawed and dogmatic as claiming that they are the primary cause of Alzheimer’s disease. Instead, its intention was to underscore the significance of building model organisms on the foundation of objective scientific facts that enable hypothesis testing, rather than designing them to manifest phenotypes aligned with a particular scientific hypothesis that, although widely accepted as factual, may lack empirical proof.

Dr. Johansson raises two significant points worth considering. The first point pertains to the BRI2-Aβ model (Kim et al, 2013). In this model, BRI2 is employed as an alternative Aβ precursor protein. Presumably, this choice is based on the widely accepted notion that pathogenic BRI2 mutations underlie AD-related dementia by generating amyloidogenic mutant peptides, specifically ADan in Familial Danish Dementia and ABri in Familial British Dementia. In this pathogenic context, the substitution of the ADan/ABri-producing region with Aβ appears logical. This perspective, akin to transgenic models that involve APP overexpression, regards the physiological functions of amyloid peptide precursors (such as BRI2 and APP) as inconsequential, with a concentrated emphasis on the central role of amyloidogenic outcomes. As Dr. Johansson aptly highlights, this approach can introduce unforeseen confounding factors, underscoring the importance of comprehending the biological functions of overexpressed proteins. Dr. Johansson reasons that the BRI2-Aβ hybrid transgene has a dual role in generating both Aβ and an anti-amyloidogenic factor, BRI2 BRICHOS, which likely contributes to the reduction of Aβ aggregation and toxicity in vivo. This plausible mechanism could provide an explanation for the lack of behavioral deficits observed in these mice. Additionally, I want to emphasize that mature BRI2 possesses supplementary anti-amyloidogenic functions. Specifically, mature BRI2 interacts with APP and consequently decreases overall APP processing, including the production of Aβ (Tamayev et al, 2012). Therefore, it is reasonable to propose that the anti-amyloid functions of both mature BRI2 and BRI2 BRICHOS prevent behavioral deficits in these animals. Consequently, it is possible that animal models solely producing human Aβ, without the simultaneous overexpression of BRI2, might exhibit functional impairment. Finally, BRI2 BRICHOS and mature BRI2 may possess additional biological functions that are possibly unrelated to the effects on amyloid fibril formation and APP processing but could still potentially contribute to mitigating the proposed toxic effects of Aβ. Some of these functions have been documented, such as the role of BRI2 in glutamatergic synaptic transmission (Yao et al, 2019). Others have been more recently described (Shimozawa et al, 2023; Yin et al, 2024) or remain undiscovered. However, despite these persuasive arguments, which incidentally underscore some of the challenges associated with transgenic models discussed in the commentary (D’Adamio, 2023), it remains an undeniable fact that amyloid plaques continue to form, and high levels of soluble Aβ oligomeric species accumulate in the brains of BRI2-Aβ mice (Kim et al, 2013). These findings appear to be at odds with the expected toxicity of Aβ. Is it possible to make a quantitative argument in this regard? Certainly, one could contend that higher levels of pathogenic Aβ forms might be necessary to induce behavioral deficits. Yet, it is perhaps useful to interpret the results from the BRI2-Aβ mice in conjunction with the data from APPsi:tTA transgenic mice, that show how improvement in cognitive performance and network hyperexcitability of APP overexpressing mice correlates with decrease in levels of full-length APP, soluble APP ectodomains, and APP C-terminal fragments, but not amyloid load, soluble, insoluble, and oligomeric Aβ42.

Dr. Johansson raises another important argument. He notes that “treatment of APP^NL-F^ and APP^NL-G-F^ knock-in mice, which overproduce Aβ40 and Aβ42 but no other APP processing intermediates, with intravenous rh Bri2-BRICHOS injections improves memory function along with reduced plaque load” (Manchanda et al, 2023). The evidence that Bri2-BRICHOS improves memory of APP^NL-F^ and APP^NL-G-F^ knock-in mice, and that this effect correlates with the estimated reduction of Aβ42 oligomer generation and neurotoxicity, presents a strong argument, albeit correlative, in favor of the notion that Aβ plays a pivotal role in the memory deficits observed in these mice. However, a few reflections are worth considering.

Given the physical and functional interaction between mature BRI2 and APP, it is conceivable that Bri2-BRICHOS could potentially modify this interaction and/or influence the function of soluble APP ectodomains. It is unclear whether these possibilities have been investigated and ruled out.

APP carrying the NL mutation (the Swedish mutation) is cleaved more efficiently by β-secretase and less efficiently by α-secretase as compared to wild-type APP (Citron et al, 1992; Tambini et al, 2019). Thus, the primary effect of these mutations is an increase in the direct metabolites of APP cleavage by BACE/β-secretase (soluble APPβ and APP-βCTF), and a reduction of the direct metabolites of APP cleavage by α-secretase (soluble APPα and APP-αCTF). In this context, it is worth highlighting that cleavage of APP by BACE/β-secretase within glutamatergic synaptic vesicles appears to finely upregulate the release probability of these vesicles by disruption of an intra-vesicular functional domain of APP. The evidence indicating that augmenting BACE/β-secretase cleavage of APP, as seen with the Swedish mutation, results in an elevated release probability of glutamatergic synaptic vesicles, supports the hypothesis that this cleavage, in and of itself, has functional consequences linked to the disruption of the intra-vesicular functional domain of APP (Tambini et al, 2019). Furthermore, there is evidence to suggest that APP-βCTF may have a pathological role that is distinct from its role as an Aβ precursor (Im et al, 2023; Tamayev et al, 2012). The Aβ rise caused by the Swedish mutation is secondary to the increase in the availability of APP-βCTF, the APP metabolite from which Aβ is generated upon γ-secretase cleavage. Thus, it is predictable that APP cleavage intermediates are profoundly altered in both APP^NL-F^ and APP^NL-G-F^ mice.

In their remarkable paper (Manchanda et al, 2023), Johansson and colleagues demonstrate that Bri2-BRICHOS also alleviates astrogliosis and microgliosis in both APP^NL-F^ and APP^NL-G-F^ mice. While these effects could be, as proposed, secondary to the anti-amyloid function of Bri2-BRICHOS, they may also involve distinct mechanisms, potentially through direct modulation of astrocyte and microglia activities. In this regard, it is noteworthy that *ITM2B* is expressed across all CNS cell types, with microglia exhibiting significantly higher levels of *ITM2B* mRNA expression than other brain cell types, while astrocytes rank as the second highest expressors (Yin et al, 2024). Moreover, BRI2 plays a crucial role in microglia, influencing processes such as microglia clustering, phagocytosis, and the secretion of cytokines/chemokines (Yin et al, 2024). Intriguingly, BRI2 and TREM2, a microglia-specific protein implicated in late-onset Alzheimer’s Disease, interact within microglia, impacting TREM2 processing and function. Notably, Bri2-BRICHOS contains one of the two BRI2 domains that interact with TREM2, and there is a direct interaction between recombinant Bri2-BRICHOS and the soluble TREM2 ectodomain. These findings provide an empirical basis for considering that Bri2-BRICHOS treatment may have attenuated microgliosis in APP^NL-F^ and APP^NL-G-F^ mice by directly affecting Bri2 functions in microglia, as well as influencing the functions of soluble Trem2, and potentially modulating the biological effects of the Bri2-Trem2 complex in microglia. Although the function of BRI2 in astrocytes remains unexplored, it is theoretically plausible that Bri2-BRICHOS may exert a direct impact on astrogliosis by either altering or mimicking the astrocytic functions of BRI2. It is also worth considering whether Bri2-BRICHOS could impact the functioning of Bri2 at glutamatergic synapses, potentially mediating some of the beneficial effects observed. Additionally, there could be other unknown effects of Bri2-BRICHOS, possibly associated with the various Bri2-BRICHOS binding proteins identified by Johansson and colleagues (Shimozawa et al, 2023), that may contribute to the therapeutic outcomes seen in APP^NL-F^ and APP^NL-G-F^ mice.

In conclusion, the findings presented by Johansson and colleagues are both intriguing and robust. The assertion that the therapeutic benefits of Bri2-BRICHOS stem from its anti-amyloid properties is certainly plausible. However, it is equally valid to consider the possibility that Bri2-BRICHOS could exert additional effects. Therefore, it remains an open question whether the therapeutic effects of Bri2-BRICHOS are solely attributed to its anti-amyloid activity or if they might result from a combination of various factors, which could or could not encompass its anti-amyloid capabilities.
